# 877. North American Phase 3/3b Experience with Long-Acting Cabotegravir and Rilpivirine: Efficacy, Safety, and Virologic Outcomes

**DOI:** 10.1093/ofid/ofab466.1072

**Published:** 2021-12-04

**Authors:** Babafemi O Taiwo, Darrell Tan, Parul Patel, Paula Teichner, Joseph Polli, Louise Garside, Ronald D’Amico, Christine L Talarico, Rodica Van Solingen-Ristea, Bryan Baugh, William Spreen, Michael Aboud, Matthew Bosse

**Affiliations:** 1 Northwestern University, Chicago, IL; 2 Division of Infectious Diseases, Department of Medicine, St. Michael’s Hospital, Toronto, Ontario, Canada; 3 ViiV Healthcare, Research Triangle Park, NC; 4 GlaxoSmithKline, London, England, United Kingdom; 5 Janssen Research & Development, LLC, Beerse, Antwerpen, Belgium

## Abstract

**Background:**

Cabotegravir (CAB) plus rilpivirine (RPV) is the first complete long-acting (LA) regimen recommended by treatment guidelines for the maintenance of HIV-1 virologic suppression. CAB+RPV LA dosed every 4 weeks (Q4W) or every 8 weeks (Q8W) demonstrated noninferior efficacy in multinational Phase 3/3b trials. This *post hoc* descriptive analysis summarizes efficacy, virologic outcomes, safety, and treatment preference for US and Canadian (CAN) participants through Week (W) 48.

**Methods:**

This analysis focuses on data for US/CAN participants naive to CAB+RPV (n=376) from the larger pooled population of the ATLAS, FLAIR, and ATLAS-2M Phase 3/3b studies (N=1245). Endpoints included the proportion of participants with plasma HIV-1 RNA ≥ 50 and < 50 c/mL at W48 (FDA Snapshot algorithm), incidence of confirmed virologic failure (CVF; 2 consecutive HIV-1 RNA ≥ 200 c/mL), safety, and treatment preference through W48.

**Results:**

376 US/CAN participants received CAB+RPV LA Q4W or Q8W. Median (range) age was 39y (20–74); 14.9% were female, 66.0% were White. At W48, 93.1% (350/376) maintained virologic suppression (HIV-1 RNA < 50 c/mL), 1.9% (7/376) had HIV-1 RNA ≥ 50 c/mL, and 0.8% (3/376) met the CVF criterion, consistent with the overall global pooled population (Table 1). Two of the three participants with CVF had ≥ 2 of the three baseline factors (archived RPV resistance-associated mutations [RAMs], HIV subtype A6/A1, body mass index [BMI] ≥ 30 kg/m^2^) previously associated with CVF. Among the US/CAN participants with a single baseline factor, none met CVF. Overall, archived RPV RAMs were observed in 3.2% (12/376), HIV subtype A6/A1 in 1.1% (4/376), and BMI ≥ 30 kg/m^2^ in 26.3% (99/376) of participants. Safety and injection site reaction findings were similar to the overall pooled population (Table 2). Most participants (120/134, 89.6%) preferred LA over oral dosing (7/134, 5.2%).

Table 1. Snapshot outcomes following CAB+RPV LA Q4W and Q8W at Week 48 in participants naive to CAB+RPV from ATLAS, FLAIR, and ATLAS-2M (ITT-E population)

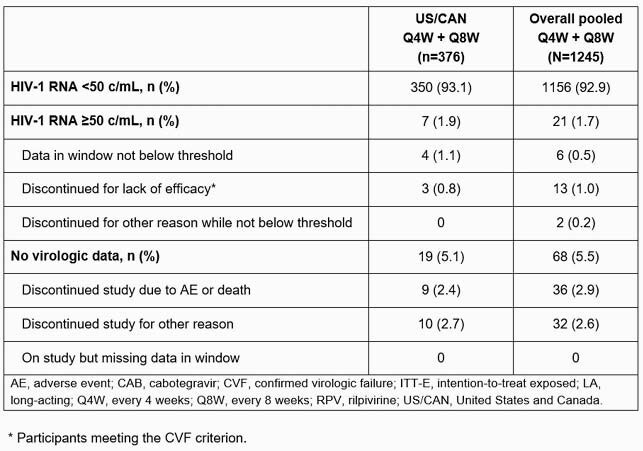

Table 2. Safety summary through Week 48 following CAB+RPV LA Q4W and Q8W or comparator ART in participants naive to CAB+RPV from ATLAS, FLAIR, and ATLAS-2M

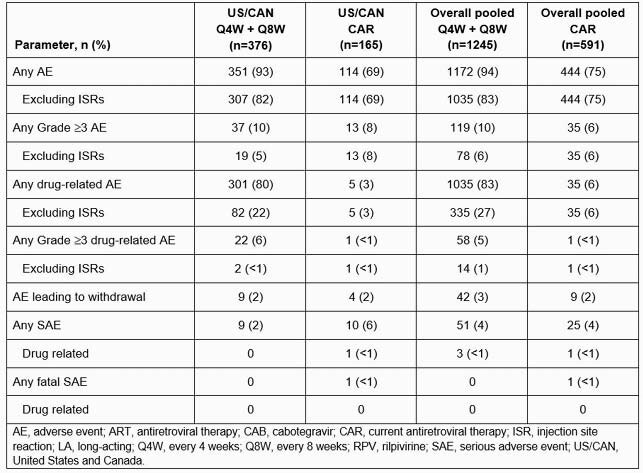

**Conclusion:**

In US/CAN Phase 3/3b trial participants, CAB+RPV LA was highly effective and well tolerated, with outcomes consistent with the overall pooled population. Baseline prevalence of archived RPV RAMs and subtype A6/A1 was low and aligned with regional prevalence/surveillance data. CAB+RPV LA provides a tolerable and effective injectable LA treatment option for virologically suppressed US/CAN individuals with HIV.

**Disclosures:**

**Babafemi O. Taiwo, MBBS**, **Gilead** (Consultant)**Merck** (Consultant)**ViiV Healthcare** (Consultant) **Darrell Tan, MD PhD**, **Abbvie** (Grant/Research Support)**Gilead** (Grant/Research Support)**GlaxoSmithKline** (Scientific Research Study Investigator)**ViiV Healthcare** (Grant/Research Support) **Parul Patel, PharmD**, **GlaxoSmithKline** (Shareholder)**ViiV Healthcare** (Employee) **Paula Teichner, PharmD**, **GlaxoSmithKline** (Shareholder)**ViiV Healthcare** (Employee) **Joseph Polli, PhD, FAAPS**, **GlaxoSmithKline** (Shareholder)**ViiV Healthcare** (Employee) **Louise Garside, PhD**, **GlaxoSmithKline** (Employee) **Ronald D’Amico, DO, MSc**, **GlaxoSmithKline** (Shareholder)**ViiV Healthcare** (Employee) **Christine L. Talarico, M.S.**, **GlaxoSmithKline** (Shareholder)**ViiV Healthcare** (Employee) **Rodica Van Solingen-Ristea, MD**, **Janssen Research and Development** (Employee)**ViiV Healthcare** (Employee) **Bryan Baugh, MD**, **Janssen, Johnson & Johnson** (Employee, Shareholder) **William Spreen, PharmD**, **GlaxoSmithKline** (Shareholder)**ViiV Healthcare** (Employee) **Michael Aboud, MBChB, MRCP**, **GlaxoSmithKline** (Shareholder)**ViiV Healthcare** (Employee) **Matthew Bosse, DO**, **GlaxoSmithKline** (Shareholder)**ViiV Healthcare** (Employee)

